# Dietary Supplementation with Chestnut (Castanea sativa) Reduces Abdominal Adiposity in FVB/n Mice: A Preliminary Study

**DOI:** 10.3390/biomedicines8040075

**Published:** 2020-04-04

**Authors:** Pedro Rodrigues, Tiago Ferreira, Elisabete Nascimento-Gonçalves, Fernanda Seixas, Rui Miguel Gil da Costa, Tânia Martins, Maria João Neuparth, Maria João Pires, Germano Lanzarin, Luís Félix, Carlos Venâncio, Isabel C.F.R. Ferreira, Margarida M.S.M. Bastos, Rui Medeiros, Isabel Gaivão, Eduardo Rosa, Paula A. Oliveira

**Affiliations:** 1 Centre for the Research and Technology of Agro-Environmental and Biological Sciences (CITAB), UTAD, 5000-801 Vila Real, Portugal; pedro20jsp@hotmail.com (P.R.); tiagoterras55@gmail.com (T.F.); elisabete.nascimento.g@gmail.com (E.N.-G.); rmcosta@fe.up.pt (R.M.G.d.C.); taniam@utad.pt (T.M.); joaomp@utad.pt (M.J.P.); glanzarin@utad.pt (G.L.); lfelix@utad.pt (L.F.); cvenanci@utad.pt (C.V.); erosa@utad.pt (E.R.); 2 Department of Veterinary Sciences and Animal and Veterinary Research Centre (CECAV), University of Trás-os-Montes and Alto Douro (UTAD), Quinta de Prados, 5000-801 Vila Real, Portugal; fseixas@utad.pt; 3 Laboratory for Process Engineering Environment Biotechnology and Energy (LEPABE) Chemical Engineering Dept, University of Porto Faculty of Engineering, (FEUP), 4200-465 Porto, Portugal; mbastos@fe.up.pt; 4 Molecular Oncology and Viral Pathology Group, IPO-Porto Research Center (CI-IPOP), Portuguese Institute of Oncology of Porto (IPO-Porto), 4200-072 Porto, Portugal; 5 Postgraduate Programme in Adult Health (PPGSAD), Federal University of Maranhão (UFMA), São Luís, 65080-805 Maranhão, Brazil; 6 Research Center in Physical Activity, Health and Leisure (CIAFEL), Faculty of Sports, University of Porto, 4200-450 Porto, Portugal; mneuparth@hotmail.com; 7 CEBIMED, Faculty of Health Sciences, Fernando Pessoa University, 4249-004 Porto, Portugal; 8 Instituto de Investigação e Inovação em Saúde (i3s), Laboratory Animal Science (LAS), Universidade do Porto (UP), 4200-135 Porto, Portugal; 9 Department of Animal Science, School of Agrarian and Veterinary Sciences, University of Trás-os-Montes and Alto Douro (UTAD), 5000-801 Vila Real, Portugal; 10 Centro de Investigação de Montanha (CIMO), Instituto Politécnico de Bragança, Campus Santa Apolónia, 5300-253 Bragança, Portugal; iferreira@ipb.pt; 11 Faculty of Medicine, University of Porto (FMUP), 4200-319 Porto, Portugal; ruimmms@gmail.com; 12 LPCC Research Department, Portuguese League against Cancer (NRNorte), 4200-172 Porto, Portugal; 13 Department of Genetics and Biotechnology and Animal and Veterinary Research Centre (CECAV), University of Trás-os-Montes and Alto Douro (UTAD), Quinta de Prados, 5000-801 Vila Real, Portugal; igaivao@utad.pt

**Keywords:** in vivo, cholesterol, adipose tissue, oxidative stress

## Abstract

The production of chestnut (*Castanea sativa* Miller) is mostly concentrated in Europe. Chestnut is recognized by its high content of antioxidants and phytosterols. This work aimed to evaluate the effects of dietary chestnut consumption over physiological variables of FVB/n mice. Eighteen FVB/n male 7-month-old mice were randomly divided into three experimental groups (*n* = 6): 1 (control group) fed a standard diet; 2 fed a diet supplemented with 0.55% (*w*/*w*) chestnut; and 3 supplemented with 1.1% (*w*/*w*) chestnut. Body weight, water, and food intake were recorded weekly. Following 35 days of supplementation, the mice were sacrificed for the collection of biological samples. Chestnut supplementation at 1.1% reduced abdominal adipose tissue. Lower serum cholesterol was also observed in animals supplemented with chestnut. There were no significant differences concerning the incidence of histological lesions nor in biochemical markers of hepatic damage and oxidative stress. These results suggest that chestnut supplementation may contribute to regulate adipose tissue deposition.

## 1. Introduction

*Castanea sativa* is a species from the Fagaceae family mainly found in Mediterranean Europe [1]. This species is of great importance as it has several purposes, namely obtaining wood, tannins, and fruits (chestnuts). Each of these products has a different application [2]. Chestnut (*Castanea sativa* Miller) has been used for several centuries as a food source in rural areas of Europe [3]. Chestnuts are mostly consumed in the autumn [2], and can be eaten in many ways: Fresh, cooked, roasted, or fried [4]. Additionally, chestnuts are used to produce creams, purées, soups, and other products [5]. In the past, chestnut contributed as an important input of energy and protein in the diet of economically disadvantaged populations [6]. Chestnut is a low-fat fruit [6,7] that is rich in minerals and vitamins [3], has high levels of moisture [7] and considerable amounts of fiber, and contains high levels of starch [3,7]. Moreover, chestnut contains phospholipids, tocopherols, and sterols and fatty acids, particularly linoleic [8]. Chestnuts are low in sodium and high in potassium, phosphorus, and magnesium [9]. In addition to these properties, chestnut has tannins, although these are only present in large quantities in the red inner shell and the brown shell, which is removed during food preparation [9].

Although the chestnut has never been studied as a whole, beneficial properties for health are attributed to its compounds. For example, acid linoleic plays an important role in preventing cardiovascular disease in adults and promotes brain and retinal development in children [3]. The phospholipid content in chestnut is associated with its high antioxidant activity [10]. In vitro and animal studies suggest that γ-tocopherol has antioxidant and anti-inflammatory properties, and γ-tocotrienol has anticancer, hypocholesterolemic, and neuroprotective properties [11].

Chestnut oil is rich in polyunsaturated fatty acid (PUFAs), including linoleic and oleic acids, and tocopherols, such as γ-tocopherol, which have been associated with multiple health benefits, as previously reviewed by Ramadan [10]. Omega-3 fatty acids have been shown to prevent or ameliorate conditions with a chronic inflammatory component like Crohn’s disease, ulcerative colitis, and some autoimmune diseases, and also malignancies that are partly driven by inflammation, like breast [12], colon, and prostate [13] cancers. Diets rich in PUFAs may also contribute to improve vascular and metabolic conditions like hypertension and type 2 diabetes, and to decrease the blood concentrations of cholesterol, in particular that of low-density lipoproteins (LDLs) [14]. Additionally, chestnut oil contains large amounts of phytosterols, particularly β-sitosterol, followed by stigmasterol [8], both of which may also contribute to reduce cholesterol levels and prevent diabetes [15,16]. Actually, chestnut has the highest levels of phytosterols when compared to other nuts (almonds, hazelnuts, walnuts, and peanuts) [5]. Considering all these findings, chestnuts seem to be a healthy component of the human diet, especially for celiac, diabetic, and high-cholesterol patients; in addition, chestnuts seem to reduce the risk of coronary heart diseases [17].

Taking into account these multiple reports on the hypocholesterolemic effects of the chestnut components, this study aimed to evaluate the physiological and potential toxicological changes associated with two different chestnut concentrations (0.55% and 1.1%, *w*/*w*) included in the diet of FVB/n mice.

## 2. Materials and Methods

### 2.1. Sample Preparation

A maintenance diet, a commercial rodent feed (consisting, by energy, of 53.5% of carbohydrate, 3% of fat, 18.5% of protein, and 5% of fiber; Mucedola 4RF21 Certificate, Milan, Italy), was used as a basis for the preparation of modified diets containing raw chestnut without the inner and the outer shell (AgroAguiar, Agroindústria SA, Sabroso de Aguiar, Portugal). The edible chestnuts were reduced to flour in a food processor and incorporated into two different modified diets and concentrations of 0.55%. and 1.1% (*w*/*w*). These concentrations were calculated assuming that, during the chestnut season, an adult (70 kg) person consumes 150 g per serving or 450 g per week. For a 30-g mouse with a 5-g average daily food intake, this corresponds to 192.6 mg of chestnuts/week/mouse and the 0.55% (*w*/*w*) diet. The high-intake diet (1.1% *w*/*w*) was conceived to double the edible chestnut intake. The diets were prepared using an industrial mixer (CPM Europe, C-300 model, Zaandam, The Netherlands) and adding 5% (*v*/*w*) water to the mix to form new pellets (4.2 mm in diameter). The edible chestnut inclusion possibly added more carbohydrates and fiber but also fatty acids, phenolic compounds, and organic acids to the commercial feed. The base diet was prepared in the same method but without chestnut incorporation. All food lots were subsequently dried in an oven at 40 °C for 48 h and stored at 4 °C until further use.

### 2.2. Experimental Procedures

All experimental procedures were approved by the University of Trás-os-Montes and Alto Douro Ethics Committee (10/2013) and the Direção Geral de Alimentação e Veterinária (0421/000/000/2014). The animals were kept under controlled conditions of temperature (23 ± 2°C), light-dark cycle (12 h light/12 h dark), and relative humidity (50 ± 10%). Eighteen FVB/n 7-month-old male mice (*Mus musculus*) were randomly divided into three different groups (n=6/group): Group 1, control group fed the standard diet without edible chestnut supplementation; group 2, fed a diet supplemented with 0.55% chestnut; and group 3, supplemented with 1.1% of chestnut. The well-being of the animals was checked weekly as well as the body weight of each animal and the water and food consumption. The animals were sacrificed 35 days after the beginning of the experimental procedures by intraperitoneal administration of ketamine (Imalgene 1000, Ventóquinol, Barcarena, Portugal) and xylazine (Rompun^®^ 2% Bayer, Healthcare S.A., Kiel, Germany), followed by cardiac puncture and exsanguination according to FELASA guidelines. Complete necropsies were performed. Heart, lungs, spleen, liver, thymus, kidneys, and abdominal and perirenal fat were collected and weighed in a precision balance (KERN ^®^PLT 6200-2A, Dias de Sousa S.A., Alcochete, Portugal).

#### 2.2.1. Hematology

Blood samples were centrifuged in capillary tubes at 4500 × g for 5 min and microhematocrit values were obtained. For biochemical analyses, heparinized blood was centrifuged at 1400 × g for 15 min and plasma was stored at −80°C until further use. The concentrations of alanine aminotransferase (ALT), aspartate aminotransferase (AST), creatinine, and cholesterol were determined by spectrophotometric methods using an autoanalyzer (Prestige 24i, Cormay PZ).

#### 2.2.2. Comet Assay

The alkaline (pH > 13) comet assay was performed in mononuclear blood cells. A system of eight gels per slide was adopted by Guilherme et al. [18], in order to increase the yield. Briefly, 4 slides precoated with normal melting point agarose were prepared per mouse. Blood was diluted in 200 µL of ice-cold phosphate-buffered saline (PBS) and 20 μL of this cell suspension were mixed with 70 μL of 1% low melting point agarose. Eight drops were placed onto the 4 precoated slide (2 replicates per slide). The samples were incubated with a lysis solution (2.5 M NaCl, 0.1 M EDTA, 10 mM Tris, 1% Triton X-100, pH 10) at 4 °C, for 1 h and rinsed (40 mM HEPES, 0.1 M KCl, 0.5 mM EDTA, 0.2 mg/mL bovine serum albumin, pH 8.0). In order to specifically measure oxidative damage to DNA, namely 8-oxoguanines and other altered purines, 2 slides were incubated with formamidopyrimidine DNA glycosylase (FPG), which converts oxidized purines into DNA single-strand breaks. The enzyme was generously donated by Professor Andrew Collins (University of Oslo, Oslo, Norway). Then, slides with and without FPG treatment were incubated in an alkaline electrophoresis solution (0.3 M NaOH and 1 mM EDTA for 30 min at 4 °C) and electrophoresed for 30 min, at 25 V and 300 mA. The cells were then neutralized with PBS followed by distilled water, dehydrated in 70% and absolute ethanol. DNA was stained with 4,6-diamidino-2-phenylindole (DAPI) and visualized using a fluorescent microscope (OLYMPUS R XC10, U-RFL-T, Hamburg, Germany). By the visual method, the comets were classified according to the tail intensity (0 class—without damage and 4 class—high damage) [19]. The total score expressed as a genetic damage indicator (GDI) was calculated according to the formula:

GDI = [(% nucleoids class 0)×0] + [(% nucleoids class 1)×1] + [(% nucleoids class 2)×2] + [(% nucleoids class 3)×3] + [(% nucleoids class 4)×4].

One hundred comets (50 comets per gel) were scored to obtain a genetic damage index (GDI) on a scale ranging between 0 and 400 arbitrary units. Scores obtained with FPG incubation (GDI_FPG_) were subtracted from the untreated GDI to quantify net enzyme-sensitive sites (NSS_FPG_).

#### 2.2.3. Hepatic and Kidney Histology

Liver and kidney samples were fixated in 10% neutral buffered formalin and embedded in paraffin. Tissue sections (2-μm-thick) were stained with hematoxylin and eosin for observation under optical microscopy and histological analysis. In hepatic tissues, the presence of mitotic figures, intracellular inclusions, binucleated hepatocytes, tri- or multinucleated hepatocytes, focal necrosis and inflammation, cells with morphological changes suggestive of apoptosis, and hepatocellular vacuolar degeneration were recorded. Renal lesions were classified as non-suppurative interstitial nephritis, isolated cell necrosis, cell changes compatible with chronic nephropathy/tubular regeneration, and tubular accumulation of protein casts suggestive of proteinuria.

#### 2.2.4. Hepatic and Kidney Oxidative Stress

Liver and kidney samples were collected and stored at −80 °C until they were processed for oxidative stress analysis. The samples were homogenized in cold buffer solution (0.32 mM of sucrose, 20 mM of HEPES, 1 mM of MgCl_2_, and 0.5 mM of phenylmethylsulfonylfluoride (PMSF), prepared in ethanol to prevent protein degradation, pH 7.4), centrifuged (15,000× *g* for 20 min at 4°C) (Sigma model 3K30, Osterode, Germany), and supernatants were collected. Total superoxide dismutase (SOD) activity was estimated according to the method described by Durak et al. [20] The activity of catalase (CAT) was estimated at 240 nm by a method previously described [20] using bovine catalase as a standard (0–5 U/mL). Gluthathione S-Transferase (GST) activity was estimated due to the reaction of the thiol group of glutathione with 1-chloro-2,4-dinitrobenzene (CDNB), analyzing the increase in absorbance at 340 nm. A molar extinction coefficient of 9.60 mM-1 cm-1 was used. The ration between reduced glutathione (GSH) and oxidized glutathione (GSSG) was determined as the oxidative-stress index (OSI). The reactive oxygen species (ROS) synthesis was estimated by using a 2,7-dichlorofluorescein diacetate (DCFDA) probe, with excitation at 485 nm and emission at 530 nm as previously described [21]. An indicator of lipid peroxidation (LPO), malondealdehyde (MDA) was determined by the thiobarbituric acid (TBA)-based method [22].

#### 2.2.5. Statistical Analysis

Body weight gain was calculated as previously [23]. Statistical analysis was performed using IBM SPSS version 25 (Statistical Package for the Social Sciences Chicago, Illinois, EUA). The data obtained were used to calculate means and standard errors. The data were analyzed using ANOVA test followed by Bonferroni’s test to test whether differences between groups were statistically significant (*p* < 0.05).

## 3. Results

### 3.1. General Findings

During the experimental work, no phenotypic or behavioral changes were observed among the experimental mice. Additionally, no mortality was observed during the experimental period. The mean body weight increased throughout the test in all groups and ponderal weight gains were not statistically different (*p* > 0.05, Table 1).

Mice supplemented with edible chestnut consumed significantly (*p* < 0.05) more food than controls (group 1), but there were no significant differences concerning water consumption (Table 2). Group 3 (1.1% chestnut) presented significantly lower abdominal adipose tissue weights compared with groups 1 (control) and 2 (0.55% chestnut) (*p* < 0.005) as shown in Table 3. There were no significant differences concerning other organ weights.

### 3.2. Hematology

The microhematocrit values were not statistically different between groups (Table 4). Group 1 (control) mice showed higher total cholesterol values compared with groups 2 (0.55% chestnut) and 3 (1.1% chestnut), but the difference was not significant.

### 3.3. Liver and Kidney Histology

Results from the renal and hepatic histology are summarized in Table 5. Again, there were no significant differences between groups.

### 3.4. Comet Assay

The genetic damage index (GDI) scores obtained from the comet assay without FPG were not statistically different between groups (Table 6). When the assay was performed following incubation with FPG to detect potential oxidative DNA damage to peripheral blood leukocytes, the differences in GDI_FPG_ scores between groups were also not significant.

### 3.5. Liver and Kidney Oxidative Stress

Concerning hepatic and renal oxidative stress analyses, no significant differences were observed between groups for any markers (*p* > 0.05) (Table 7).

## 4. Discussion

This is the first experimental study to evaluate the effects of dietary supplementation with different edible chestnut concentrations in mice (*Mus musculus*). Noh and colleagues previously evaluated the effects of the chestnut inner shell (*Castanea crenata*) on HepG2 (human liver cancer cell line) cells and in C57BL/6 mice treated with a high-fat diet. Inner shell extracts showed the ability to reduce oxidative damage caused by tert-butylhydroperoxide in HepG2 cells in vitro, as well as oxidative damage caused by carbon tetrachloride [24]. Jovanović and co-workers found that spiny burrs extract of sweet chestnut (*Castanea sativa* Mill) improved the liver and kidney function in diabetic Wistar rats, by reducing oxidative damage towards lipids and DNA and inhibiting protein glycation [25]. However, none of these previous studies addressed the effects of chestnut kernels in the diet in such a way as to reproduce the values of chestnuts consumed by humans.

This experiment is a preliminary study, so we used a small sample of animals (calculated using the power of analysis) in order to achieve results and, at the same time, apply the 3Rs (Replacement, Reduction and Refinement) principle predicted for animal experiments. During the present experimental work, no mortality was recorded in any group and no physiological or behavioral changes were observed, supporting the idea that edible chestnut supplementation is well tolerated. In line with these findings, all groups showed positive ponderal weight gains. Experimental groups supplemented with edible chestnuts showed significantly higher feed intake, suggesting that the chestnut was highly palatable. Importantly, this increased food intake did not result in significant weight differences or obesity. Other studies from our group in which the diet of FVB/n mice was similarly manipulated with the addition of the polyphenols curcumin and rutin showed similar results [26]. There were no significant differences in the relative weight of internal organs between groups, again suggesting that edible chestnut supplementation was safe in this experimental setting. A study from Nyengaard and colleagues concluded that kidney weight is positively correlated with the number of glomeruli present and the size of the glomeruli [27]. Jovanović found that diabetic Wistar rats presented a distorted renal architecture with glomerulopathy and tubular degenerescence and that treatment with sweet chestnut extract partially preserved the renal architecture [25]. In line with these observations, in the present study, we did not observe significant histological or oxidative stress changes associated with edible chestnut supplementation, even at the higher dosage of 1.1%. Creatinine values were lower in the groups supplemented with edible chestnut, although the difference was not significant. Lower blood plasma creatinine levels in animals from groups 2 (0.55% chestnut) and 3 (1.1% chestnut) may be associated with a more efficient glomerular filtration rate.

The values obtained in the microhematocrit did not present statistically significant differences between the groups, in line with previous findings from another group [28]. There were also no significant changes concerning hepatic transaminases, ALT, and AST, suggesting that dietary supplementation with chestnut did not induce hepatotoxicity at the biochemical level. This is in line with the results of Jovanović et al. [25], who administered spiny burrs extracts of sweet chestnut to Wistar rats and did not observe hepatic toxicity. The results from histological and oxidative stress analyses performed in hepatic tissues agree with the blood biochemistry, indicating that the edible chestnut supplementation was safe in these experimental conditions.

The most interesting finding from the present study is the lower relative weights of peri-renal and abdominal adipose tissue in mice supplemented with 1.1% chestnut. This may be explained by the amount of digestion-resistant starch in chestnut (*C. sativa*), which accounts for over 50% of the total starch content in this kind of nut [29], and also by its high PUFAs content. Si and co-workers concluded that resistant starch significantly reduces the weight of adipose tissue in Wistar rats with a high-fat diet [30]. Chestnut supplementation also increased the concentration of polyunsaturated and monounsaturated fatty acids in the adipose tissue of pigs [31]. Monounsaturated fatty acids and omega-3 PUFAs present in adipose tissue are indirectly associated with obesity [32]. These findings are significant, as reduced body fat contributes to improving metabolic and vascular health, preventing conditions like diabetes and atherosclerosis [33], and also inflammation-associated conditions and certain types of cancer [12,13]. Groups supplemented with edible chestnut also showed a trend towards lower total blood cholesterol values, reinforcing the potential of chestnut to protect metabolic and vascular homeostasis. These results may be explained by the presence of PUFAs, namely linoleic acid, which plays a key role in cholesterol reduction [33]. Alongside linoleic acid, the γ-tocotrienol and sterols present in chestnuts also have an impact on cholesterol reduction [10,11]. A previous study in Zucker *fa*/*fa* rats (obesity rat model)concluded that omega-3 PUFAs led to a reduction in cholesterol levels [34]. Another study also found that chestnut spiny burrs extracts decreased cholesterol levels in diabetic Wistar rats [25].

The comet assay did not reveal significant increases in DNA damage in animals supplemented with edible chestnut, even when samples were pre-treated with FPG to reveal oxidative DNA-damage. In fact, there was a trend towards reduced oxidative DNA damage in animals that received edible chestnut. Based on these results, dietary supplementation with edible chestnut does not seem to cause damage to DNA and it may even reduce oxidative DNA damage, in line with the findings of Jovanović et al. [25], who showed that spiny burrs extracts of sweet chestnut reduce DNA damage in the liver and kidney cells of diabetic rats. This protective effect may be associated with the antioxidant properties of phenolic compounds in chestnuts [35]. Extracts of the chestnut inner shell (*C. crenata*) reduced oxidative stress in C57BL/6 mice exposed to a high-fat diet [24]. Similar results were reported by Grdović et al. [35], who observed that spiny burrs extract of sweet chestnut (*C. sativa Mill*) reduced DNA damage induced by streptozotocin in pancreatic β-cells, and associated this reduction with the high content of ellagic acid and its derivatives in chestnut. In fact, ellagic acid was already known to reduce DNA damage in Chinese hamster ovary cells through comet assay and cytofluorimetric analyses [36].

We intend to carry out additional studies with higher doses of chestnut, different exposure times, and other types of chestnut preparations. In addition, our team intends to carry out an obesity model to assess the effects of chestnut on the animals’ body weight, cholesterol levels, and the percentage of white and brown fat.

## 5. Conclusions

These experimental works suggest that an edible chestnut diet can be beneficial by reducing visceral fat and total blood cholesterol. Moreover, no toxic effects were observed in association with dietary edible chestnut supplementation, even when using doses estimated to be twice higher than those generally consumed by human populations.

## Figures and Tables

**Table 1:** Body weight variation (g) throughout the experimental work and weight gain (mean ± standard error).

	Group 1 (No Chestnut *n* = 6)	Group 2 (0.55% Chestnut *n* = 6)	Group 3 (1.1% Chestnut *n* = 6)
**0 Week (g)**	32.54 ± 0.73	33.63 ± 3.20	32.45 ± 2.54
**1 Week (g)**	33.70 ± 0.81	34.55 ± 2.83	33.92 ± 3.18
**2 Week (g)**	34.04 ± 0.86	34.85 ± 2.69	34.48 ± 3.34
**3 Week (g)**	34.35 ± 0.90	35.09 ± 2.95	34.39 ± 2.70
**4 Week (g)**	33.96 ± 0.69	35.58 ± 2.75	34.90 ± 3.08
**5 Week (g)**	34.04 ± 0.78	35.55 ± 3.09	33.94 ± 3.06
**Weight Gain (g)**	0.044 ± 0.024	0.055 ± 0.014	0.047 ± 0.027

**Table 2:** Mean daily food and water consumption per animal at experimental weeks 1 and 5.

	Group 1 (No Chestnut *n* = 6)	Group 2 (0.55% Chestnut *n* = 6)	Group 3 (1.1% Chestnut *n* = 6)
**Food (g)**			
**1st week**	4.30	4.59	4.99 *
**5th week**	3.98	4.63 *	4.89 *
**Water (mL)**			
**1st week**	4.70	5.10	6.02
**5th week**	4.27	6.00	6.03

* Statistically different from group 1 (control) (*p* < 0.05).

**Table 3:** Relative organ weights, peri-renal, and abdominal adipose tissue (mean ± standard error).

	Group 1 (No Chestnut *n* = 6)	Group 2 (0.55% Chestnut *n* = 6)	Group 3 (1.1% Chestnut *n* = 6)
**Heart (g)**	0.0047± 0.00014	0.0048 ± 0.00043	0.0047 ± 0.00087
**Lung (g)**	0.0053± 0.00049	0.0058 ± 0.00040	0.0060 ± 0.00103
**Spleen (g)**	0.0035± 0.00102	0.0033 ± 0.00130	0.0033 ± 0.0043
**Liver (g)**	0.0487± 0.00400	0.0517 ± 0.00317	0.0469 ± 000395
**Thymus (g)**	0.0011 ± 0.00046	0.0010 ± 0.00050	0.0014 ± 0.00043
**Right kidney (g)**	0.0076 ± 0.00100	0.0082 ± 0.00102	0.0084 ± 0.00109
**Left kidney (g)**	0.0076 ± 0.00067	0.0079 ± 0.00151	0.0079 ± 0.00101
**Peri-renal fat (g)**	0.0050 ± 0.00245	0.0072 ± 0.00288	0.0044 ± 0.0148
**Abdominal fat (g)**	0.0198 ± 0.00358 *	0.0199 ± 0.00513 *	0.0112 ± 0.00364

* Statistically significant difference from group 3 (*p* < 0.05).

**Table 4:** Microhematocrit (Ht) and serum biochemical parameters (mean ± standard error).

	Group 1 (No Chestnut *n* = 6)	Group 2 (0.55% Chestnut *n* = 6)	Group 3 (1.1% Chestnut *n* = 6)
**Ht (%)**	41.27 ± 1.621	41.34 ± 0.303	41.93 ± 3.360
**Creatinine (mg/dL)**	0.342 ± 0.282	0.120 ± 0.088	0.332 ± 0.309
**Cholesterol (mg/dL)**	157.85 ± 4.10	139.68 ± 6.29	151.88 ± 25.89
**AST (U/L)**	65.17 ± 21.43	49.42 ± 14.06	66.86 ± 29.82
**ALT (U/L)**	43.37 ± 6.32	34.56 ± 8.22	34.44 ± 9.01

**Table 5:** Histological classification of liver and kidney lesions in mice.

Experimental Groups	Group 1 (No Chestnut *n* = 6)	Group 2 (0.55% Chestnut *n* = 6)	Group 3 (1.1% Chestnut *n* = 6)
Liver lesions			
Mitotic cells	2/6 (33.3%)	1/6 (16.6%)	2/6 (33.3%)
Intracellular Inclusions	1/6 (16.7%)	4/6 (66.7%)	4/6 (66.7%)
Tri- or multinucleated hepatocytes	1/6 (16.6%)	1/6 (16.6%)	3/6 (50.0%)
Focal necrosis and inflammation	2/6 (33.3%)	3/6 (50.0%)	2/6 (33.3%)
Apoptosis	0 (0.0%)	1/6 (16.6%)	2/6 (33.3%)
Vacuolar degeneration	0 (0.0%)	0 (0.0%)	1/6 (16.6%)
Kidney lesions			
Non-suppurative interstitial nephritis	3/6 Focal (50.0%)	6/6 multifocal (100.0%)	6/6 multifocal (100.0%)
Protein casts	0 (0.0%)	1/6 (16.7%)	1/6 (16.7%)
Isolated cell necrosis	2/6 (33.3%)	4/6 (66.7%)	4/6 (66.7%)
Chronic nephropathy/regeneration	1/6 (16.7%)	2/6 (33.3%)	0 (0.0%)

**Table 6:** Genetic damage index (GDI) with and without formamidopyrimidine DNA glycosylase (FPG) determined by comet assay (mean ± standard error).

Experimental Groups	Group 1 (No Chestnut, *n* = 6)	Group 2 (0.55% Chestnut, *n* = 6)	Group 3 (1.1% Chestnut, *n* = 6)
GDI	43.42 ± 7.44	50.83 ± 25.64	44.00 ± 15.84
GDIFPG	44.30 ± 10.42	31.33 ± 7.53	33.50 ± 10.46

**Table 7:** Oxidative stress parameters evaluated in the liver and kidney of mice (mean ± standard deviation).

Oxidative Stress Parameters	Group 1 (No Chestnut *n* = 6)	Group 2 (0.55% Chestnut *n* = 6)	Group 3 (1.1% Chestnut *n* = 6)
**Liver**			
**ROS** (µmol DCF mg ^−1^ protein)	396 ± 166.9	371.6 ± 52.79	339.9 ± 142.6
**SOD** (U mg ^−1^ of protein)	418.4 ± 171.7	376.2 ± 193.5	904.7 ± 534.3
**CAT** (U mg ^−1^ of protein)	84.14 ± 31.55	61.52 ± 19.91	46.2 ± 37.33
**GST** (nmol CDNB/min.mg^-1^ of protein)	147 ± 70.51	137.7 ± 22.1	196.7 ± 38.33
**OSI**	1.01 ± 0.84	1.09 ± 0.47	0.77 ± 0.36
**LPO** (µmol MDA mg ^−1^ of protein)	29.8 ± 9.69	33.86 ± 4.53	31.7 ± 11.19
**Kidney**			
**ROS** (µmol DCF mg ^−1^ protein)	396.2 ± 223.2	409.1 ± 210.2	363.7 ± 133.2
**SOD** (U mg ^−1^ of protein)	406.4 ± 222.7	312.3 ± 122.5	532.8 ± 330.5
**CAT** (U mg ^−1^ of protein)	57.11 ± 22.26	43.03 ± 3.95	44.52 ± 18.56
**GST** (nmol CDNB/min.mg ^−1^ of protein)	20.13 ± 5.34	12.08 ± 16.13	17.39 ± 9.67
**OSI**	1.50 ± 1.51	2.64 ± 1.88	1.52 ± 0.72
**LPO** (nmol MDA mg ^−1^ of protein)	33.62 ± 10.5	41.48 ± 17.41	48.83 ± 12.96
